# Antagonism to Plant Pathogens by *Epichloë* Fungal Endophytes—A Review

**DOI:** 10.3390/plants10101997

**Published:** 2021-09-24

**Authors:** Stuart D. Card, Daniel A. Bastías, John R. Caradus

**Affiliations:** 1 AgResearch Limited, Grasslands Research Centre, Private Bag 11008, Palmerston North 4442, New Zealand; daniel.bastias@agresearch.co.nz; 2 Grasslanz Technology Limited, Private Bag 11008, Palmerston North 4442, New Zealand; john.caradus@grasslanz.com

**Keywords:** antifungal activity, biological control, cool-season grass, *Lolium*, pooideae, symbiosis

## Abstract

*Epichloë* is a genus of filamentous fungal endophytes that has co-evolved with cool-season grasses with which they form long-term, symbiotic associations. The most agriculturally important associations for pasture persistence for grazing livestock are those between asexual vertically transmitted *Epichloë* strains and the pasture species, perennial ryegrass, and tall fescue. The fungus confers additional traits to their host grasses including invertebrate pest deterrence and drought tolerance. Selected strains of these mutualistic endophytes have been developed into highly efficacious biocontrol products and are widely utilized within the Americas, Australia, and New Zealand for pasture persistence. Less publicized is the antagonism *Epichloë* endophytes display towards multiple species of saprophytic and pathogenic microbes. This opinion piece will review the current literature on antimicrobial properties exhibited by this genus of endophyte and discuss the reasons why this trait has historically remained a research curiosity rather than a trait of commercial significance.

## 1. Introduction

Most multicellular life on Earth lives in symbiosis with microorganisms [1]. Plants, for example, whether they are growing within natural or managed ecosystems, are constantly interacting with a myriad of living microorganisms, including archaea, bacteria, fungi, and protists, throughout their lifecycle [2]. This microbial community (the plant microbiota) coupled with the surrounding environment (the entire habitat = the microbiome) has distinct physio-chemical properties and is crucially important for the health and productivity of the host plant. The interactions between hosts and microbes, or symbiosis, are complex ranging from mutualism through commensalism to parasitism in a continuous manner [3,4] and are important ecological determinants of plant biodiversity [5], although some regard parasitism as an unbalanced status of the symbiosis [6]. Furthermore, mutualistic symbioses were likely responsible for early host plant habitat transitions in the late Precambrian era (ca. 600 Ma) with several microbial species implicated in the evolution of photosynthesizing organisms [7,8,9,10]. This concept forms the basis for the hologenome theory of evolution [11] and proposes that the holobiont, the plant host plus all of its symbionts, is a unit of selection [12]. Although the first mutualistic symbioses were likely to have been associations between simple monerans and protists that led to the Cambrian explosion [10], present day symbiosis are more diverse. Examples include lichens (associations between algae or cyanobacteria and several fungal species), those between rhizobia and legumes, mycorrhiza (those associations between plants and fungi), and associations between *Epichloë* fungal endophytes and cool-season grasses.

## 2. *Epichloë* Endophytes

*Epichloë* (family Clavicipitaceae) is a monophyletic genus of filamentous fungi that form perpetual symbioses with cool-season grasses (family Poaceae subfamily Pooideae) [13]. These endophytes are regarded as keystone species, being ecologically important constituents of many grassland ecosystems, which cover over 30% of the Earth’s land area and are, therefore, one of the largest biomes on the planet [14,15,16]. *Epichloë* species are naturally restricted to a host genus or closely related grass genera within a tribe as a result of co-evolution over many millennia [17,18,19]. However, this group of grass symbionts may have originally emerged from an animal pathogen via an interkingdom host jump [20,21]. As with most members of the Ascomycota, the genus *Epichloë* includes both anamorphic (asexual) and teleomorphic (sexual) species, with the former previously classified as *Neotyphodium* [22]. Over 30 species of *Epichloë* have been described [22] with most exhibiting an anamorphic lifecycle.

Obligatory sexual species of *Epichloë* are largely antagonistic to their host plant as the epiphytic stroma that is formed on the developing host inflorescence, essential for horizontal transmission, suppresses plant seed production, thereby impeding the hosts ability to reproduce [23]. In contrast, the asexual species are symptomless within their host plants being exclusively vertically transmitted via the host’s seed [24,25]. Many of the plant’s progeny inherit chromosomes and cytoplasm from their parents but also their mutualistic symbionts, constituting a form of hereditary endosymbiosis [26]. *Epichloë* have been documented to confer a multitude of beneficial attributes to their host grasses. These attributes differ across the various endophyte–host associations with the most prominent being protection from mammalian and invertebrate herbivory via the production of secondary metabolites, most notably alkaloids [27]. The major metabolites involved in this defensive mutualism include peramine, an insect feeding deterrent; the lolines, a group of saturated *exo*-1-amino pyrrolizidines that exhibit a broad spectrum of insecticidal activity; indole diterpenes and ergot alkaloids that contain classes of compound that exhibit toxicity towards both vertebrates and invertebrates [28]. Additional attributes include tolerance against abiotic stresses (i.e., when the plant is exposed to adverse environmental conditions) such as those caused by drought and/or nutrient deficiencies [29,30].

*Epichloë* endophytes were initially identified as the cause of a serious agriculture issue due to some endophyte-grass associations producing alkaloidal neurotoxins that are detrimental to many animal species, including livestock (i.e., farmed ruminants) [31]. The most potent of these compounds include the lolitrems (indole diterpenes) and ergot alkaloids that result in ryegrass staggers and heat stress/fescue-foot syndromes respectively [32,33,34]. Research on the *Epichloë* endophyte associations with *Lolium perenne* L. (perennial ryegrass) in New Zealand (NZ) and *Lolium arundinaceum* (Schreb.) S.J. Darbyshire (= *Festuca arundinacea* Schreb.; tall fescue) in the USA, identified asexual *Epichloë* strains that were less toxic to livestock whilst conferring advantageous traits to their host grasses. Since this initial research in the 1980s and 1990s, scientific programmes have been established that center on bioprospecting pipelines to identify, characterize and select agriculturally beneficial endophyte strains (those that confer advantageous traits to their host while producing little or no detrimental effects to grazing livestock) that could be incorporated into elite grass cultivars with increased pasture persistence and productivity [30,31].

AgResearch (a NZ government owned research institute that carries out scientific research for the benefit of NZ; https://www.agresearch.co.nz; accessed on 15 September 2021) developed bioprospecting pipelines that (1) identify *Epichloë* endophytes from global collections of germplasm, (2) characterize *Epichloë* isolates with respect to their genetic diversity, secondary metabolite profiles and bioactivity, (3) inoculate agriculturally useful strains with commercial potential into elite grass cultivars, and (4) evaluate populations of novel grass-endophyte associations through specifically designed agronomic and toxicological screens [35,36]. Endophyte strains that are devoid of lolitrems and ergot alkaloids while possessing insect-deterring compounds (e.g., peramine and/or lolines) generally have commercial potential. This opinion piece will review the current literature on the antimicrobial properties exhibited by *Epichloë* fungal endophytes and discuss the reasons why this trait has historically remained a research curiosity rather than a trait of commercial significance.

## 3. Interaction of *Epichloë* spp. with Plant Pathogens

Although a great deal of knowledge regarding the bioactivity of *Epichloë*-derived secondary metabolites on invertebrate pests has been gained, little research has been undertaken on the biological control of fungal phytopathogens by *Epichloë*-infected grasses [37,38]. To the best of our knowledge, no published research documents the biological control of bacterial phytopathogens by *Epichloë,* although certain endophyte strains can influence the host plant’s bacterial microbiota [39]. Furthermore, no correlation exists between the antifungal bioactivity expressed by these endophytes and their alkaloid profiles [40,41] and although several *Epichloë*-based grass products are marketed across the continents of Australia, North America and South America for their insect deterrent properties [30,31,42], none are marketed for their control of plant diseases. However, *Epichloë* endophytes can inhibit the growth and reproduction of other microorganisms, including economically important fungal phytopathogens [43]. A recent meta-analysis has shown that all species of phytopathogen significantly affected by the presence of *Epichloë* endophyte regardless of their lifestyle (biotrophic vs. necrotrophic), with both laboratory and greenhouse comparisons showing negative effects of *Epichloë* spp. on the growth and infection by phytopathogens [44]. However, many of the published reports describe bioactivity that has been assessed using crudely designed in vitro dual culture bioassays (Table 1), with fewer reports on this phenomenon in planta within a field situation (Table 2).

Biological control, in its simplest form, can be defined as applied ecology [88] and is an environmentally sound and effective means of reducing or mitigating viral, microbial, nematode, insect, mite, weed, and vertebrate pests in agriculture, aquatic, forest, natural resources, stored products, and urban environments. Many primary screens using artificial nutrient media are severely criticized as they do not closely resemble the final arena where biological control ultimately takes place. Subsequently, these primary screens, which can determine antagonism between an endophyte and a phytopathogen in vitro, are generally poor at predicting protection within live plant tissues [89,90]. For example, in vitro research conducted in Finland showed clear antifungal activity expressed by strains of *Epichloë* towards the speckled snow mold pathogen, *Typhula ishikariensis*, when grown on potato dextrose agar (PDA). However, in the subsequent field experiment, the endophyte-infected grasses were more susceptible to the pathogen than the endophyte-free control plants [63].

This lack of correlation between in vitro and in planta results may be due to multiple reasons: (1) some *Epichloë*-derived secondary metabolites are only produced in planta. For example, the endophyte-derived alkaloids are metabolites only produced by the grass-endophyte association with the exception of two loline alkaloids that can be produced in vitro from axenic endophyte cultures at a relatively low concentration from a defined minimal media [91], (2) concentrations of *Epichloë*-derived secondary metabolites depend on the host plant. These endophytes may not be able to secure the relevant nutrients or amount of nutrients in planta and the secondary metabolite biosynthetic pathways may be down regulated as with some biotrophic phytopathogens [92], and (3) the in vitro bioassays are largely restricted to identifying antibiosis as the sole mechanism of action exhibited by these fungi while other mechanisms exist. In vitro screens can, therefore, be misleading by either overestimating or underestimating the potential of endophyte strains to protect against phytopathogens.

Antibiosis is defined as antagonism mediated by specific or non-specific metabolites of microbial origin, by lytic agents, enzymes, volatile compounds or other toxic substances [88]. However, there are additional mechanisms of biological control proposed for *Epichloë*-plant associations [93], including induced resistance (reduced disease susceptibility of a plant in response to stimulation by a pathogen, insect herbivore, beneficial microbe, or chemical agent [94]) and competition for limiting factors (e.g., physical space, carbohydrates and amino acids) (see Table 2 for examples). The fourth main mechanism of biological control, direct parasitism, exhibited by several fungal antagonists such as *Trichoderma* spp. [95], has not been reported to date for *Epichloë* spp. The endophyte would have to engage in direct contact with the target phytopathogen, and this seems unlikely due to the restriction of these endophytes within the intracellular spaces of their grass hosts and the sometimes-suppressive nature of the regulation that they encounter during their growth and development [96,97].

As mentioned previously, no correlation exists between the antifungal bioactivity expressed by these endophytic fungi and their alkaloid profiles [40,41]. However, several non-alkaloid secondary metabolites have been implicated in the antagonism exhibited by *Epichloë* spp. towards phytopathogens (see antibiosis studies listed in Table 2). These compounds include sesquiterpenes [45,98], phenolic glycerides [99], hydroxyl unsaturated fatty acids [100], aromatic sterols [101], indole derivatives (indole-3-acetic acid (IAA) and indole-3-ethanol), diacetamides [45] and other volatile insect-attractant compounds, such as Chokol K and methyl esters [102]. Chokol K is an interesting compound, hypothesized to of originated as an antimicrobial agent the compound also has the ability to attract *Botanophila* flies, fungal pollinators of the external fruiting structures, the stroma, of sexually reproducing *Epichloë* species [103].

Research conducted on the bioactivity of *Epichloë* endophytes (Card, unpublished) aligns with previous studies in the literature that show that many *Epichloë* species exhibit antifungal activity towards a wide range of saprophytic and pathogenic fungal species, including those with diverse taxonomy (e.g., species from both the Ascomycota and Basidiomycota) and those with different lifestyles (e.g., biotrophs and necrotrophs) (Table 1 and Table 2). This could indicate that several antifungal compounds are produced simultaneously or that a small number of antifungal compounds have broad antifungal activity. Further work showed that no antifungal activity was attributed to guttation fluid collected from perennial ryegrass plants infected with selected *Epichloë* strains (Lambie and Christensen, unpublished) similar to that found with peramine against insects [104]. This may indicate that if antifungal compound/s are solely responsible for biological control within this system then they may have limited mobilization within the plant.

The *Epichloë*-mediated induction of the plant’s own defenses has been proposed as another mechanism of resistance against phytopathogens [43,44]. Plant defenses are regulated by hormonal signaling pathways, including salicylic acid (SA) and jasmonic acid (JA) [105]. The dominant model for conceptualizing plant defenses suggests that the SA and JA signaling pathways protect plants against biotrophic and necrotrophic pathogens, respectively [106]. *Epichloë* spp. can activate host plant SA- and JA-signaling pathways [107]. This *Epichloë*-based activation of plant defense hormonal signaling pathways may explain, at least in part, the increased levels of disease resistance exhibited by *Epichloë*-infected plants (see IR studies listed in Table 2). For example, enhanced plant resistance exhibited by endophyte-infected *A. inebrians* plants against the biotrophic pathogen *B. graminis* was related to the activation of SA signaling pathways, increment in SA levels, and the upregulation in the expression of SA-related genes coding for putative plant enzymes with antifungal activities (i.e., β-1,3-glucanase and callose synthase) [77]. Similarly, the enhanced plant resistance exhibited by *A. sibiricum* plants against the necrotrophic pathogen *C. lunata* by *A. sibiricum* was correlated to increased levels of JA and plant phenolics [79].

Further novel mechanisms have been proposed for specific grass-endophyte combinations, for example niche exclusion has been proposed for *Epichloë* associations with *Bromus setifolius*, *Festuca ovina*, *F. rubra*, and *Poa ampla* [108]. This mechanism results in the exclusion of phytopathogens by a superficial network of endophyte mycelium that develops on the leaf blade surface of grasses. The epiphytic mycelium was hypothesized to be defensive in function to physically exclude the entry of fungal pathogens into the leaves [109]. Pérez et al. [44] further proposed that two classes of mechanism exist with respect to the reduction of plant disease by *Epichloë* spp., (1) direct pathways mediated by antibiosis, competition and IR (described above) and (2) indirect pathways associated with endophyte-generated changes in either the abiotic or the biotic host environment. These authors describe an indirect pathway for the protection of *L. multiflorum* by its fungal endophyte *E. occultans* from the flower-infecting pathogen *Claviceps purpurea*, the causal agent of ergot [44]. The incidence and severity of *C. purpurea* infection was two-fold lower in endophyte-symbiotic plants than in non-symbiotic ones but when insects were prohibited from visiting the flowers this difference disappeared, indicating that endophyte-derived volatile compounds repel insect vectors of *C. purpurea* and indirectly defend their host grasses against plant disease [80].

Antagonism between *Epichloë* spp. and phytopathogens may have evolved many millions of years ago. A recent article describes an ancient cross-kingdom gene transfer (the transfer of genetic material between organisms) from *Epichloë* to tall wheatgrass (*Thinopyrum ponticum*). The article suggests that Fhb7, a major, semidominant resistance gene, was transferred to the wild cereal grass around 5 million years ago after the divergence of *Thinopyrum* from other grasses [110]. Fhb7 encodes a glutathione S-transferase that detoxifies deoxynivalenol (DON), a mycotoxin that renders grain poisonous to humans and other mammals. It is speculated that *Epichloë* may have evolved Fhb7 to compete with *Fusarium* spp. for grass colonization [111]. Furthermore, transcriptomic studies have identified an abundantly expressed fungal gene coding for a small, secreted protein, similar to antifungal proteins found within species of *Penicillium* and *Aspergillus*, in *Epichloë festucae*-infected red fescue plants [112]. The antifungal protein gene is not found within the genomes of many other *Epichloë* spp. and is hypothesized to be a component of the unique disease resistance observed with endophyte-infected red fescue plants to dollar spot disease caused by the phytopathogen *Clarireedia jacksonii* [113]. It is suggested that once the endophyte lifestyle evolved in *Epichloë*, and the wider Clavicipitaceae endophytes, the alkaloid and other fungal–mediated defensive features adapted from previous functions to serve as plant host defense functions [114].

Recent theoretical modelling studies suggested that in order to predict biocontrol outcomes there is a need to understand not only the main biocontrol mechanisms involved but also the extent of environmental variability, the level of biocontrol activity, and survival of individual biological control agents in relation to external conditions [115]. *Epichloë* fungi, and other mutualistic endophytes, have a distinctive advantage over other biocontrol microorganisms that are inoculated into the phyllosphere or rhizosphere through drenches or sprays. By inhabiting the intracellular spaces of their plant hosts for their entire lifecycle they are buffeted from many cyclic and non-cyclic variables, including potentially damaging ultraviolet rays, extremes of temperature, humidity, dew, rain and wind as experienced, for example, on the leaf surface [88]. Endophytic microorganisms are also protected from faster proliferating, more competitive microorganisms as experienced in many phyllosphere and rhizosphere environments. Endophyte survival is firmly tied with that of the plant host and are therefore totally reliant on the plant for substrates (e.g., water, sugars, and oxygen) and a suitable ecological niche free from competing microorganisms, along with the absence of inhibitory or toxic substances. While nutrients may become limiting in the phyllosphere environment, endophytes are supplied with a bountiful array of organic and inorganic nutrients including sugars, sugar alcohols, nitrates, nitrites, amino acids, organic acids, calcium, chloride, phosphorus, potassium, sulfur and soluble proteins [as reviewed by 116] supplied to the apoplasm from the neighboring phloem. Although the concentration and availability of such compounds within the apoplasm may fluctuate, it is largely a copiotrophic environment [116]. Substrates are not only in plentiful supply to support endophyte growth (biomass) but also may support the production of many secondary metabolites, including many anti-mammalian and insect deterrent alkaloidal compounds [28] as well as antimicrobial compounds active against phytopathogens.

The inhibition of phytopathogens by *Epichloë* has, however, largely remained a research curiosity rather than an attribute exploited for commercial disease biocontrol purposes as very few in planta experiments document the control of economically important phytopathogens in the field. These may be due to several reasons:

(1) Many phytopathogens, particularly foliar fungal pathogens, are notoriously difficult to control (even by conventional synthetic agrichemicals) as they can produce vast numbers of wind-disseminated spores that are spread over large distances over significant periods of time. These phytopathogens can also exhibit a polycyclic nature that can be completed in just a few days, continuously barraging the plant with fresh inoculum.
(2) Many plant diseases occur due to underlying abiotic disorders (e.g., nutrient imbalance, and/or water stress) that have weakened or stressed the plant and made it susceptible to invasion by a phytopathogen. Additionally, disease in the field is generally brought about by a complex of interacting microorganisms including primary and secondary pathogens making identification of the causal organism difficult.
(3) Bioprospecting pipelines have to date identified *Epichloë* strains with deterrent traits against economically important invertebrate pests and therefore endophyte strains with high levels of bioactivity towards phytopathogens could have been missed, overlooked, or not prioritised.
(4) Many in vitro primary screens overestimate the bioactivity of selected endophyte strains and do not screen enough strains to identify those with high potential for commercialisation. Great strain variation exists with respect to antifungal activity [53] and even morphotypes of the same species from the same host grass species can vary in their antifungal activity [37]. Furthermore, host genotype × endophyte effects can impact the degree of antifungal activity expressed.
(5) A lack of understanding concerning the mechanisms of action attributed to selected *Epichloë* strains. These mechanisms need to be understood to develop suitable novel grass-endophyte associations.
(6) Many end users completely ignore the concept of biological control in favour of agrichemicals while others view it as essentially a compete synthetic chemistry replacement. However, biological control products have been generally less reliable than agrichemicals which has hampered their development, release, and commercial uptake [117]. For example, *Epichloë* strains with antifungal activity will generally only reduce the frequency or size of diseased lesions and, therefore, disease is still present [53].

## 4. Interaction of *Epichloë* Endophytes with Other Taxa

As well as fungal phytopathogens, *Epichloë*, or their metabolites, can influence the interactions between endophyte-infected plants and other organisms. As discussed, endophyte-derived alkaloids can influence invertebrates and species such as herbivorous mammals and this in turn can affect species diversity and fundamental ecological processes like decomposition, and food web structures [118,119,120,121,122]. In natural ecosystems, *Epichloë* can reduce plant diversity, enhance the dominance of certain grass-endophyte associations [5] and also generate legacy effects which persist after the death of the host [123]. *Epichloë* endophytes may also impact the microbiome (the microbiota and its theatre of activity) of the vegetative host plant effecting microbial community structure in above and below ground habitats [121]. Studies aimed at investigating the rhizosphere communities of tall fescue plants show that *Epichloë*-infected plants showed a higher species richness over endophyte-free rhizospheres and a greater percentage of Firmicutes [124] while the presence of *Epichloë gansuensis* within its host grass *Achnatherum inebrians* significantly decreased root-associated fungal diversity [125]. These findings within the rhizosphere are paralleled within the phyllosphere where some endophyte-infected fescue species select particular epiphytic bacterial microbiota [39] with loline alkaloids implicated as a significant carbon source for certain bacterial genera including *Burkholderia*, *Serratia*, *Pseudomonas* and *Stenotrophomonas* [124]. In the rhizosphere, strains of these bacterial taxa have subsequently been shown to outcompete and suppress the growth of non-loline catabolizing strains [124]. *Epichloë* endophytes can also impact plant reproduction with endophyte-infected seed harbouring higher populations of plant-growth promoting bacteria compared to endophyte-free varieties, with these bacteria possibly playing an important role in the fitness of the subsequent seedlings [126]. Further work is looking at how *Epichloë* endophytes may shape entire ecological communities [15,127].

A substantial amount of research has investigated the interaction between *Epichloë* species and well-known beneficial microorganisms, such as arbuscular mycorrhizal (AM) fungi [128]. AM fungi aid the host by supplying mineral nutrients (mostly phosphorus) improving the nutritional status of colonised plants while also increasing the host’s tolerance to certain abiotic and biotic stresses [4,129]. In planta studies investigating the interaction between *Epichloë* endophytes and AM fungi show that the amount by which symbiotic plant roots are colonised by AM fungi depends on the type of grass-*Epichloë* association [130]. For example, agronomic, novel grass-*Epichloë* associations often have lower amounts of mycorrhizal mycelia in roots of symbiotic plants (i.e., artificial symbioses: *L. perenne*-*E. festucae* var. *lolii*, *Lolium multiflorum*-*Epichloë occultans*, and *F. arundinacea*-*E. coenophiala*) while wild grasses infected with their co-evolved *Epichloë* endophytes usually display greater abundances of AM fungi in roots of symbiotic plants (i.e., *Bromus* spp. and *Poa* spp.) [131,132,133,134,135,136,137,138,139,140]. Further complexities also exist with regard to species interactions [141,142]. *Epichloë*-derived bioactive compounds, competition for nutrients, and/or changes in the levels of plant resistance to microorganisms have been proposed as mechanisms to explain the interaction between *Epichloë* and AM fungi [137].

Recent research from NZ has also determined that *Epichloë* strains can associate closely with specific bacterial species [143] and may work in synergy to antagonise microorganisms that threaten the survival and reproduction of the host plant. This has been shown in other tripartite interactions within multiple fungal species [144] with bacterial endosymbionts responsible for mycotoxin and phytohormone production including IAA [145,146]. IAA is the major plant auxin and is also synthesized by certain microorganisms, including phytopathogens, saprophytes and symbionts [147]. IAA stimulates the production of plant biomass, enhances growth rate of roots, and is implicated in the promotion of disease resistance [147,148]. Tripartite associations involving *Epichloë* endophytes are not restricted to bacteria with several articles reporting that *Epichloë* spp. can also be infected with mycoviruses [149,150,151]. Although these viruses appear to have no effect on the phenotype of their fungal hosts, the fungus, or plant infected by the endophytic fungus and the virus, may obtain selective advantages yet to be discovered.

## 5. Future Perspectives

Climate change, the most important challenge currently facing mankind [152] is predicted to have serious implications for many agricultural systems. The interaction between disease and crops is as old as agriculture itself [153] but with increasing temperatures and changing precipitation patterns, it is expected that plant disease outbreaks may intensify in some production areas due to invasions of new phytopathogens and/or due to increases in the severity of existing phytopathogens [154,155,156]. Increasing atmospheric CO2 levels will impact the degree of resistance exhibited by many plant species to pathogen attack while also altering the availability of photosynthates and defensive compounds produced by plant-associated microorganisms, including *Epichloë* [66,157,158]. For example, when CO2 was artificially elevated, the beneficial effect of *Epichloë* on its tall fescue host was lost with respect to plant growth and pathogen resistance towards *C. lunata* [66]. Other gases involved in climate change, such as tropospheric ozone, have also been shown to have detrimental effects on plant host fitness and the concentration of *Epichloë* -derived defense compounds [159].

Further challenges include improving and/or changing current agricultural practices that are not sustainable as they can expend valuable resources while degrading the environment [160]. Many agrichemicals have negative effects on the environment due to overuse and inefficient application [161] while the control of plant diseases using synthetic pesticides raises serious concerns about food safety, environmental quality and pathogen resistance [162]. Coupled with other pastoral management practices (e.g., tillage), this has also led to a decrease in soil biodiversity [163] in many regions worldwide. Integrating knowledge from both agricultural and natural ecosystems, from single plants and multispecies plant communities, and from below-ground and above-ground multitrophic interactions holds great promise to further improve the sustainability of crop production, including the need for alternative disease management practices [164].

While *Epichloë* endophytes are not naturally found in modern cereal grasses it has been demonstrated that *Epichloë* strains from wild cereal grass relatives [165] can be inoculated into barley (*Hordeum vulgare*), rye (*Secale cereale*) and wheat (*Triticum aestivum*) to create artificial plant-endophyte associations [166,167,168]. Field trials with *Epichloë*-infected rye have shown reductions in the prevalence of leaf rust (*Puccinia recondita*) and leaf streak (*Cercosporidium graminis*) [65]. The potential for other fungal endophyte taxa to be used as biological control agents against phytopathogens in rice, wheat and maize has been proposed as a worthy research aim [169].

## 6. Concluding Remarks

Microorganisms have been administered as biological control agents for many decades to manage disease and pest pressures on crop plants. However, they still only make up a small percentage of all pest control products. As well as their overall bioactivity and efficacy in the field, many other factors (such as their stability, reliability, storage and application) must be taken into consideration for the development of commercially successful biological control agents [170]. Endophytes can overcome many of the difficulties faced by traditional biological control agents as they are encapsulated within the host plant and protected from environmental conditions that disrupt their survival and biocontrol efficacy. Additionally, for those biocontrol agents that are seed transmitted, such as *Epichloë* endophytes, there is an extra advantage for commercialisation as there is no need to develop complicated formulations and delivery techniques [171]. Biological control of phytopathogens is often achieved by the artificial introduction of antagonistic microorganisms into a selected environment. These antagonists may exhibit several mechanisms of action that work in synergy to suppress any one phytopathogen at any one time and although *Epichloë*-derived antibiosis may be overrepresented in many in vitro experiments this mechanism may still play a pivotal role in the protection of grass hosts from phytopathogens. Although *Epichloë* endophyte colonization is generally restricted to the host’s aboveground tissues, their bioactivity (at least towards invertebrate herbivores, via antibiosis) extends further, as many endophyte-derived alkaloids are mobilized within the plant’s vascular system and translocated to plant organs, such as the roots, where fungal colonization is absent [119,172]. Even volatile secondary metabolites derived from *Epichloë* endophytes have been reported in the roots of endophyte-infected plants [173,174].

Therefore, primary in vitro bioassays, aimed at assessing endophytes for their antagonism towards phytopathogens, must be developed to provide more reliable predictions of field performance [175]. The results from these improved bioassays, coupled with a greater understanding of the mechanisms of action attributed to these *Epichloë* endophytes, will likely lead to the development of ecologically sound and commercially viable *Epichloë*-grass associations with pest and disease control abilities.

## Figures and Tables

**Table 1:** In vitro bioactivity exhibited by species of *Epichloë* towards fungal saprophytes or phytopathogens. Bioassays reviewed here are generally dual culture assays using viable fungal colonies, or their filtered crude extracts, undertaken on potato dextrose agar (PDA) or a similar solid agar medium. These in vitro bioassays are restricted to only assessing antibiosis and cannot assess other mechanisms of action (i.e., induced resistance or competition).

Fungal Species ^1^	Host Species	Fungal Pathogen ^2^	Division	Country	Reference/s
*Epichloë amarillans*	*Achnatherum sibiricum*	*Cochliobolus lunatus* (syn. *Curvularia lunata*)	Ascomycota	China	[37]
		*Cladosporium cucumerium*	Ascomycota	China	[37]
		*Fusarium oxysporum*	Ascomycota	China	[37]
		*Phomopsis vexans*	Ascomycota	China	[37]
		*Rhizoctonia solani*	Basidiomycota	China	[37]
	*Agrostis* sp.	*Cryphonectria parasitica*	Ascomycota	USA	[45]
*Epichloë bromicola*	*Hordeum brevisubulatum*	*Alternaria* sp.	Ascomycota	China	[46]
*Epichloë chisosa*	*Achnatherum sibiricum*	*Cochliobolus lunatus* (syn. *Curvularia lunata*)	Ascomycota	China	[37]
		*Cladosporium cucumerium*	Ascomycota	China	[37]
		*Fusarium oxysporum*	Ascomycota	China	[37]
		*Phomopsis vexans*	Ascomycota	China	[37]
		*Rhizoctonia solani*	Basidiomycota	China	[37]
*Epichloë coenophialum* (FaTG-1)	*Lolium arundinaceum*	*Alternaria alternata*	Ascomycota	USA	[47]
		*Ceratobasidium cornigerum* (syn. *Rhizoctonia cerealis*)	Basidiomycota	NZ and USA	[40,48]
		*Cladosporium cladosporioides*	Ascomycota	USA	[47]
		*Cochliobolus lunatus* (syn. *Curvularia lunata*)	Ascomycota	Poland	[49]
		*Cochliobolus sativus* (syn. *Bipolaris sorokiniana*)	Ascomycota	China and Poland	[49,50]
		*Cryphonectria parasitica*	Ascomycota	USA	[45]
		*Glomerella graminicola* (syn. *Colletotrichum graminicola*)	Ascomycota	NZ and USA	[40]
		*Laetisaria roseipellis* (syn. *Limonomyces roseipellis*)	Basidiomycota	NZ and USA	[40]
		*Pyrenophora erythrospila* (syn. *Drechslera erythrospila*)	Ascomycota	NZ and USA	[41,51]
		*Gibberella acuminata* (syn. *Fusarium acuminatum*)	Ascomycota	China	[50]
		*Rhizoctonia solani*	Basidiomycota	Poland	[49]
		*Waitea circinate* (syn. *Rhizoctonia zeae*)	Basidiomycota	NZ and USA	[40]
*Epichloë* FaTG-2	*Lolium arundinaceum*	*Pyrenophora erythrospila* (syn. *Drechslera erythrospila*)	Ascomycota	NZ	[41]
		*Waitea circinate* (syn. *Rhizoctonia zeae*)	Basidiomycota	NZ	[41]
*Epichloë* FaTG-3	*Lolium arundinaceum*	*Ceratobasidium* sp.	Basidiomycota	Australia	[52]
		*Drechslera* sp.	Ascomycota	Australia	[52]
		*Pyrenophora erythrospila* (syn. *Drechslera erythrospila*)	Ascomycota	NZ	[41]
		*Waitea circinate* (syn. *Rhizoctonia zeae*)	Basidiomycota	NZ	[41]
*Epichloë festucae*	*Festuca longifolia*	*Ceratobasidium cornigerum* (syn. *Rhizoctonia cerealis*)	Basidiomycota	NZ and USA	[40]
		*Glomerella graminicola* (syn. *Colletotrichum graminicola*)	Ascomycota	NZ, Japan and USA	[40,53]
		*Laetisaria roseipellis* (syn. *Limonomyces roseipellis*)	Basidiomycota	NZ and USA	[40]
		*Pyrenophora erythrospila* (syn. *Drechslera erythrospila*)	Ascomycota	NZ	[41]
		*Waitea circinate* (syn. *Rhizoctonia zeae*)	Basidiomycota	NZ	[41]
	*Festuca pulchella*	*Botrytis cinerea*	Ascomycota	Japan	[54]
		*Cochliobolus sativus* (syn. *Bipolaris sorokiniana*)	Ascomycota	Japan	[53]
		*Drechslera dictyoides*	Ascomycota	Japan	[53]
		*Drechslera siccans*	Ascomycota	Japan	[53]
		*Glomerella graminicola* (syn. *Colletotrichum graminicola*)	Ascomycota	Japan	[53]
		*Pyrenophora erythrospila* (syn. *Drechslera erythrospila*)	Ascomycota	Japan	[53,54,55]
		*Phytophthora infestans*	Oomycota	Japan	[54]
	*Festuca rubra * ^3^	*Clarireedia homoeocarpa* (syn. *Sclerotinia homoeocarpa*)	Ascomycota	USA	[56]
		*Cochliobolus sativus* (syn. *Bipolaris sorokiniana*)	Ascomycota	Poland	[57]
		*Ceratobasidium cornigerum* (syn. *Rhizoctonia cerealis*)	Basidiomycota	NZ and USA	[40]
		*Cryphonectria parasitica*	Ascomycota	USA	[45]
		*Drechslera dictyoides*	Ascomycota	Poland	[57]
		*Glomerella graminicola* (syn. *Colletotrichum graminicola*)	Ascomycota	NZ, Japan and USA	[40,53]
		*Laetisaria roseipellis* (syn. *Limonomyces roseipellis*)	Basidiomycota	USA	[40]
		*Pyrenophora erythrospila* (syn. *Drechslera erythrospila*)	Ascomycota	NZ	[41]
		*Waitea circinate* (syn. *Rhizoctonia zeae*)	Basidiomycota	NZ	[41]
	*Festuca trachyphylla*	*Botrytis cinerea*	Ascomycota	Japan	[54]
		*Pyrenophora erythrospila*	Ascomycota	Japan	[54]
		*Phytophthora infestans*	Oomycota	Japan	[54]
	*Lolium pratense*	*Glomerella graminicola* (syn. *Colletotrichum graminicola*)	Ascomycota	Japan	[53]
*Epichloë festucae* var. *lolii* (=LpTG-1)	*Lolium perenne*	*Ceratobasidium cornigerum* (syn. *Rhizoctonia cerealis*)	Basidiomycota	NZ and USA	[40,47,48]
		*Glomerella graminicola* (syn. *Colletotrichum graminicola*)	Ascomycota	NZ and USA	[40,58]
		*Ceratobasidium* sp.	Basidiomycota	Australia	[52]
		*Cryphonectria parasitica*	Ascomycota	USA	[45]
		*Drechslera andersenii*	Ascomycota	Germany	[59]
		*Drechslera poae*	Ascomycota	Germany	[59]
		*Drechslera siccans*	Ascomycota	Germany	[59]
		*Drechslera* sp.	Ascomycota	Australia	[52]
		*Drechslera teres*	Ascomycota	Germany	[59]
		*Fusarium* sp.	Ascomycota	Australia	[52]
		*Gibberella acuminata* (*Fusarium acuminatum*)	Ascomycota	China	[50]
		*Pyrenophora erythrospila* (syn. *Drechslera erythrospila*)	Ascomycota	NZ	[41]
		*Waitea circinate* (syn. *Rhizoctonia zeae*)	Basidiomycota	NZ and USA	[40,41]
		*Laetisaria roseipellis* (syn. *Limonomyces roseipellis*)	Basidiomycota	NZ and USA	[40]
*Epichloë gansuensis*	*Achnatherum inebrians*	*Alternaria alternata*	Ascomycota	China	[50]
		*Cochliobolus lunatus* (syn. *Curvularia lunata*)	Ascomycota	China	[50]
		*Cochliobolus sativus* (syn. *Bipolaris sorokiniana*)	Ascomycota	China	[50]
		*Gibberella acuminata* (syn. *Fusarium acuminatum*)	Ascomycota	China	[50]
*Epichloë hybrida* (=LpTG-2)	*Lolium perenne*	*Pyrenophora erythrospila* (syn. *Drechslera erythrospila*)	Ascomycota	NZ	[41]
		*Waitea circinate* (syn. *Rhizoctonia zeae*)	Basidiomycota	NZ	[41]
*Epichloë* LpTG-3	*Lolium perenne*	*Ceratobasidium* sp.	Basidiomycota	Australia	[52]
		*Drechslera* sp.	Ascomycota	Australia	[52]
		*Fusarium* sp.	Ascomycota	Australia	[52]
*Epichloë occultans*	*Lolium multiflorum*	*Gibberella acuminata* (syn. *Fusarium acuminatum*)	Ascomycota	Argentina	[60] *
		*Rhizoctonia solani*	Basidiomycota	Argentina	[60] *
Unknown *Epichloë* spp.	*Festuca simensis*	*Alternaria alternata*	Ascomycota	China	[61]
		*Aspergillus niger*	Ascomycota	China	[62]
		*Cochliobolus sativus* (syn. *Bipolaris sorokiniana*)	Ascomycota	China	[61,62]
		*Cochliobolus lunatus* (syn. *Curvularia lunata*)	Ascomycota	China	[61,62]
	*Festuca versuta*	*Ceratobasidium cornigerum* (syn. *Rhizoctonia cerealis*)	Basidiomycota	USA	[48]
	*Lolium perenne*	*Glomerella graminicola* (syn. *Colletotrichum graminicola*)	Ascomycota	NZ	[58]
		*Pyrenophora erythrospila* (syn. *Drechslera erythrospila*)	Ascomycota	NZ	[58]
	*Poa ampla*	*Cryphonectria parasitica*	Ascomycota	USA	[45]
	*Poa autumnalis*	*Cryphonectria parasitica*	Ascomycota	USA	[45]
	*Poa interior*	*Cryphonectria parasitica*	Ascomycota	USA	[45]
	*Poa palustris*	*Cryphonectria parasitica*	Ascomycota	USA	[45]
	*Poa rigidifolia*	*Cryphonectria parasitica*	Ascomycota	USA	[45]
	*Poa* sp.	*Cryphonectria parasitica*	Ascomycota	USA	[45]
	*Poa sylvestris*	*Cryphonectria parasitica*	Ascomycota	USA	[45]
*Neotyphodium starii * ^4^	*Festuca arizonica*	*Glomerella graminicola* (syn. *Colletotrichum graminicola*)	Ascomycota	NZ	[40]
		*Laetisaria roseipellis* (syn. *Limonomyces roseipellis*)	Basidiomycota	NZ and USA	[40]
		*Waitea circinate* (syn. *Rhizoctonia zeae*)	Basidiomycota	NZ	[40]
	*Festuca obtusa*	*Waitea circinate* (syn. *Rhizoctonia zeae*)	Basidiomycota	NZ	[40]
*Epichloë tembladerae*	*Festuca argentina*	*Cryphonectria parasitica*	Ascomycota	USA	[45]
	*Poa hueca*	*Cryphonectria parasitica*	Ascomycota	USA	[45]
	*Poa* sp.	*Cryphonectria parasitica*	Ascomycota	USA	[45]
*Epichloë uncinatum*	*Lolium pratense* (syn. *Festuca pratensis*)	*Cochliobolus sativus* (syn. *Bipolaris sorokiniana*)	Ascomycota	Poland	[57]
		*Drechslera dictyoides*	Ascomycota	Poland	[57]
		*Gibberella avenacea* (*Fusarium avenaceum*)	Ascomycota	Poland	[57]
		*Fusarium culmorum*	Ascomycota	Germany	[59]
		*Fusarium equiseti*	Ascomycota	Poland	[57]
		*Pyrenophora erythrospila* (syn. *Drechslera erythrospila*)	Ascomycota	NZ	[41]
		*Rhizoctonia solani*	Basidiomycota	Poland	[57]
		*Typhula ishikariensis*	Basidiomycota	Finland	[63]
		*Waitea circinate* (syn. *Rhizoctonia zeae*)	Basidiomycota	NZ	[41]

^1^ Current scientific name. ^2^ Current scientific name, or most accepted name, followed by the synonym used within the citing manuscript. ^3^ Including subspecies. ^4^ *Nomen dubium* according to [22]. * In vitro experiment with axenic cultures of the pathogen and grass seed infected with *Epichloë.*

**Table 2:** In planta bioactivity exhibited by species of *Epichloë* towards fungal saprophytes or phytopathogens. In planta bioassays included all assays that did not work with axenic cultures of *Epichloë* spp. and included detached leaf assays, whole plant assays with plants grown in controlled climate rooms, glasshouses, or field trials. IR = induced resistance.

Fungal Species ^1^	Original Host Species	Type of Association	Fungal Pathogen ^2^	Division	Disease	Suggested Mechanism	Country	Reference/s
*Epichloë bromicola*	*Leymus chinensis*	original	*Cochliobolus lunatus* (syn. *Curvularia lunata*)	Ascomycota	Curvularia blight	IR	China	[64]
			*Cochliobolus sativus* (syn. *Bipolaris sorokiniana*)	Ascomycota	Spot blotch and root rot	IR	China	[64]
		original	*Waitea circinate* (syn. *Rhizoctonia zeae*)	Basidiomycota	Sheath and leaf spot	IR	Poland	[49]
	*Secale cereale*	novel	*Cercosporidium graminis*	Ascomycota	Leaf streak	Unknown	NZ	[65]
		novel	*Puccinia recondita*	Basidiomycota	Leaf rust	Unknown	NZ	[65]
*Epichloë coenophialum*	*Lolium arundinaceum*	original	*Cochliobolus lunatus* (syn. *Curvularia lunata*)	Ascomycota	Curvularia blight	Competition for nutrients	China	[66]
*Epichloë festucae*	*Festuca rubra*	original	*Cochliobolus sativus* (syn. *Bipolaris sorokiniana)*	Ascomycota	Spot blotch and root rot	Antibiosis	Poland	[57]
		original	*Dreschslera* sp.	Ascomycota	Head blight	Antibiosis	Poland	[57]
		original	*Fusarium poae*	Ascomycota	Head blight	Antibiosis	Poland	[57]
	*Festuca rubra L. subsp. rubra*	novel	*Laetisaria fuciformis*	Basidiomycota	Red thread	Competitive exclusion	USA	[67]
	*Lolium perenne*	novel	*Pyrenophora erythrospila* (syn. *Drechslera erythrospila)*	Ascomycota	Red leaf spot	Antibiosis	Japan	[53]
*Epichloë festucae* var. *lolii (=LpTG-1)*	*Lolium perenne*	original	*Alternaria alternata*	Ascomycota	Leaf spot	IR	China	[38]
		original	*Cochliobolus lunatus* (syn. *Curvularia lunata)*	Ascomycota	Curvularia blight	IR	China	[38]
		original	*Cochliobolus sativus* (syn. *Bipolaris sorokiniana)*	Ascomycota	Spot blotch and root rot	Antibiosis, competition and/or IR	China	[38,68,69]
		original	*Fusarium chlamydosporum*	Ascomycota	Wilt	IR		
		original	*Fusarium oxysporum*	Ascomycota	Fusarium wilt	IR	China	[38]
		original	*Fusarium poae*	Ascomycota	Head blight	IR	Poland	[70]
		original	*Fusarium solani*	Ascomycota	Soft root rot	IR	China	[38]
		original	*Gibberella acuminata* (syn. *Fusarium acuminatum)*	Ascomycota	Root rot	IR	China	[38]
		original	*Puccinia coronata*	Basidiomycota	Crown rust	Unknown	Australia and China	[71,72]
		novel	*Pyrenophora semeniperda*	Ascomycota	Leaf spots	Antibiosis	Australia	[73]
*Epichloë gansuensis*	*Achnatherum inebrians*	original	*Ascochyta leptospora*	Ascomycota	Ascochyta leaf blight	Antibiosis	China	[50]
		original	*Alternaria alternata*	Ascomycota	Leaf spot	Antibiosis	China	[50]
		original	*Blumeria graminis*	Ascomycota	Powdery mildew	IR	China	[74,75,76,77]
		original	*Claviceps purpurea*	Ascomycota	Ergot	IR	China	[78]
		original	*Clonostachys rosea*	Ascomycota	Unknown	Antibiosis	China	[50]
		original	*Cochliobolus lunatus* (syn. *Curvularia lunata)*	Ascomycota	Curvularia blight	Antibiosis	China	[50,79]
		original	*Fusarium chlamydosporum*	Ascomycota	Wilt	Antibiosis	China	[50]
		original	*Fusarium oxysporum*	Ascomycota	Fusarium wilt	Antibiosis	China	[50]
		original	*Fusarium solani*	Ascomycota	Soft root rot	Antibiosis	China	[50]
		original	*Gibberella acuminata* (syn. *Fusarium acuminatum)*	Ascomycota	Root rot	Antibiosis	China	[50]
		original	*Gibberella avenacea (Fusarium avenaceum)*	Ascomycota	Fusarium head blight	Antibiosis	China	[50]
		original	*Fusarium solani*	Ascomycota	Soft root rot	Antibiosis	China	[50]
	*Achnatherum sibiricum*	original	*Cochliobolus lunatus* (syn. *Curvularia lunata)*	Ascomycota	Curvularia blight	IR	China	[79]
	*Achnatherum sibiricum*	original	*Erysiphales*	Ascomycota	Powdery mildew	IR	China	[79]
*Epichloë occultans*	*Lolium multiflorum*	original	*Claviceps purpurea*	Ascomycota	Ergot	Vector exclusion	Argentina	[80]
*Epichloë sibiricum*	*Achnatherum sibiricum*	original	*Cochliobolus lunatus* (syn. *Curvularia lunata)*	Ascomycota	Curvularia blight	IR	China	[79]
	*Achnatherum sibiricum*	original	*Erysiphales*	Ascomycota	Powdery mildew	IR	China	[79]
*Epichloë sinensis*	*Festuca sinensis*	original	*Alternaria alternata*	Ascomycota	Leaf spot	Unknown	China	[81]
*Epichloë sp.*	*Bromus auleticus*	original	*Ustilago bullata*	Basidiomycota	Head smut	Unknown	Argentina	[82]
	*Festuca sinensis*	original	*Alternaria alternata*	Ascomycota	Leaf spot	Antibiosis	China	[61]
		original	*Gibberella acuminata* (syn. *Fusarium acuminatum)*	Ascomycota	Root rot	Antibiosis	China	[61]
		original	*Cochliobolus sativus* (syn. *Bipolaris sorokiniana)*	Ascomycota	Spot blotch and root rot	Antibiosis	China	[61]
		original	*Cochliobolus lunatus* (syn. *Curvularia lunata)*	Ascomycota	Curvularia blight	Antibiosis	China	[61]
	*Lolium arundinaceum*	original wild grass and cultivar	*Rhynchosporium* sp.	Ascomycota	Leaf blotch	Antibiosis, IR and/or improved host fitness	Finland	[83]
	*Lolium perenne*	original	*Drechslera siccans*	Ascomycota	Brown blight	IR	Poland	[84]
		original	*Fusarium* spp.	Ascomycota	Fusarium blight	IR	Poland	[84]
*Epichloë typhina*	*Phleum pratense*	original	*Cladosporium phlei*	Ascomycota	Purple leaf spot	Unknown	Japan	[85,86]
*Epichloë uncinatum*	*Lolium pratense (syn. Festuca pratensis)*	original	*Cochliobolus sativus* (syn. *Bipolaris sorokiniana)*	Ascomycota	Spot blotch and root rot	Antibiosis	Poland	[57]
		original	*Dreschslera* sp.	Ascomycota	Head blight	Antibiosis	Poland	[57]
		original	*Fusarium poae*	Ascomycota	Head blight	Antibiosis	Poland	[57]
		original	*Puccinia coronata*	Basidiomycota	Crown rust	Unknown	Poland	[87]

^1^ Current scientific name. ^2^ Current scientific name, or most accepted name, followed by the synonym used within the citing manuscript.

## Data Availability

All data are included in the present study.
